# Spatial and Functional Organization of the Somato-Cognitive Action Network During Cue and Execution of Motor Tasks

**DOI:** 10.21203/rs.3.rs-10638157/v1

**Published:** 2026-08-27

**Authors:** Alishba Sadiq, Jeff L. Waugh

**Affiliations:** The University of Texas Southwestern Medical Center; The University of Texas Southwestern Medical Center

**Keywords:** Effector-specific, Spatial organization, Functional organization, Task-based fMRI, Motor task, Motor homunculus

## Abstract

Primary motor cortex has traditionally been conceptualized as a somatotopically organized structure, with discrete cortical regions corresponding to specific body parts. However, accumulating evidence suggests that motor cortex organization extends beyond simple effector-based representations to include higher-order functional systems supporting goal-directed behavior. Here, we examine the somato-cognitive action network (SCAN), a set of inter-effector cortical regions located between the classical hand, foot, and face representations of primary motor cortex that has been proposed to integrate cognitive control processes with motor function. Using a fMRI motor task paradigm that dissociates cue-related and execution-related processes, we investigated the functional role and spatial organization of SCAN in healthy adults. SCAN exhibited preferential activation during the cue phase, 3.4-fold greater than during movement, indicating a prominent role in action preparation rather than motor output. In contrast, classical effector-specific motor representations (active during hand, foot, or face movement) showed greater engagement during execution, consistent with their role in movement generation. Across these SCAN regions, activation increased systematically from dorsomedial regions (adjacent to leg representations) to ventrolateral regions (adjacent to face representations). This spatial gradient was accompanied by progressively greater dominance of preparatory SCAN activity relative to movement-related activity in the adjacent effector regions. These findings demonstrate that motor cortex organization includes a distinct integrative system that is temporally and spatially dissociable from effector-specific representations. These results support an integrate–isolate framework in which cognitive and motor processes are differentially expressed across the cortical landscape, providing new insight into the functional architecture of human motor control.

## 1 Introduction

The primary motor cortex (M1) has traditionally been conceptualized as a somatotopically organized structure, based on the work by Penfield and colleagues using direct cortical stimulation (Penfield and Boldrey 1937). These studies gave rise to the classical “motor homunculus,” in which discrete cortical regions correspond to specific body parts arranged along the precentral gyrus. Although substantial overlap between representations has long been recognized (Desmurget and Sirigu 2015), this framework has remained the dominant model of motor cortex organization for nearly a century. Subsequent studies in non-human primates have revealed additional organizational features inconsistent with a simple homuncular model. For example, anatomical tracing studies have distinguished anterior “old” M1, characterized by fewer direct projections to spinal motor neurons, from posterior “new” M1, which exhibits dense corticospinal projections supporting fine motor control (Dum and Strick 1991, Rathelot and Strick 2009). Electrical stimulation experiments have further shown that limb representations can be organized into concentric functional zones, with distal effectors (e.g., digits) centrally represented and more proximal musculature arranged peripherally (Graziano, Taylor and Moore 2002). Moreover, stimulation of anterior M1 can evoke complex, multi-effector, goal-directed movements rather than isolated muscle contractions, suggesting a higher-order organizational structure (Graziano, Taylor and Moore 2002, Graziano 2006). Together, these findings indicate that M1 organization extends beyond a simple continuous somatotopic map, consistent with the idea that motor cortex may be organized around ethologically relevant action maps, in which coordinated, goal-directed behaviors, rather than individual muscles, serve as the fundamental units of organization (Graziano 2016).

While these studies highlight increasingly complex principles of motor cortex organization in non-human primates, it remains unclear how such structure is reflected in the large-scale functional organization of the human brain. Advances in human neuroimaging have enabled increasingly precise mapping of functional brain networks, including effector-specific motor circuits for the hand, face, and lower limb (Laumann, Gordon et al. 2015, Gordon, Laumann et al. 2017). Studies using precision functional mapping approaches have demonstrated that these circuits exhibit strong within-network connectivity and task-related specificity (Power, Cohen et al. 2011, Kuehn, Dinse et al. 2017). However, such effector-based representations are typically found to be relatively segregated systems that do not fully account for how motor processes are integrated with higher-order cognitive control networks to support goal-directed behavior (Greene, Marek et al. 2020, Newbold, Gordon et al. 2021). This raises the possibility that additional functional organization exists beyond classical somatotopy and effector specificity.

Although the somato-cognitive action network (SCAN) has recently been described as a distinct system within the peri-Rolandic cortex, consisting of inter-effector cortical zones located between the classical leg, hand, and face representations of primary motor and somatosensory cortex (Gordon, Chauvin et al. 2023), its functional role and internal organization remain incompletely understood. It is unclear how SCAN contributes to different phases of motor behavior, such as action preparation and movement execution, and whether its activity is uniform across its segregated nodes. The inter-effector nodes comprising SCAN are functionally and structurally distinct from the classical body-part-specific motor representations of the leg, hand, and face (hereafter referred to as effector-specific regions) and are preferentially engaged during integrative aspects of motor control (Gordon, Chauvin et al. 2023). However, the extent to which SCAN exhibits systematic spatial organization, and how its activity relates to effector-specific regions across the cortical axis, has not been established. To address these questions, we examined SCAN activity using task-based fMRI during a motor paradigm that separates cue-related and execution-related processes. We subdivided SCAN into medial, middle, and inferior regions to test whether activation varies across its cortical extent. In parallel, we analyzed effector-specific regions to directly compare integrative and movement-specific systems. Specifically, we asked: (1) Is SCAN preferentially engaged during action preparation relative to movement execution? (2) Does SCAN exhibit a graded spatial organization across the medial–lateral axis? (3) How does the relationship between SCAN and neighboring leg, hand, and face representations vary across medial, middle, and inferior cortical locations?

Our findings demonstrate that SCAN is preferentially engaged during the cue phase of motor tasks, exhibits a medial-to-inferior increase in activation during both cue and execution phases, and shows a spatially varying relationship with effector-specific regions. These results support a structured integrate–isolate framework in which the relative contributions of integrative and effector-specific processes vary across the motor cortex, providing new insight into the organization of human motor control.

## 2 Materials and Methods

**Figure 1:**
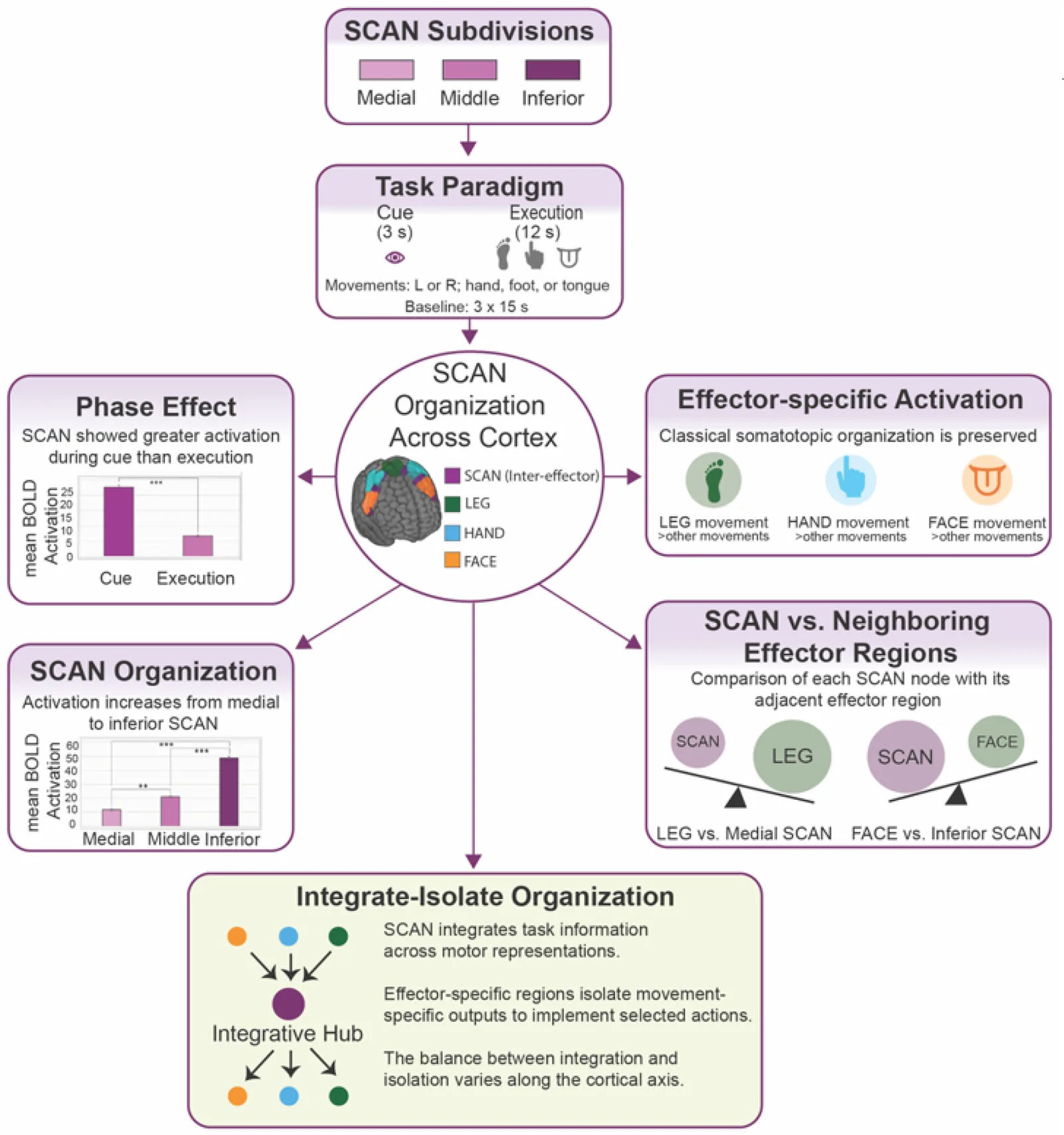
Schematic overview of the experimental framework used to investigate the spatial organization and functional role of the somato-cognitive action network (SCAN) in motor cortex. Using a motor task paradigm consisting of cue and execution phases, we first assessed phase-specific activity, demonstrating preferential engagement of SCAN during the cue phase. We analyzed effector-specific regions of primary motor cortex (leg, hand, and face) to confirm preservation of classical somatotopic organization. We subdivided SCAN into medial, middle, and inferior regions to examine potential spatial variation in activation. We evaluated the internal organization of SCAN, revealing a systematic increase in activation from medial to inferior regions. Finally, direct comparison of functional activation between each SCAN subdivision and the adjacent effector-specific motor cortex demonstrated a shift in relative dominance across the cortical axis, with effector-dominant activity in medial regions and SCAN-dominant activity in inferior regions. Together, these analyses support an integrate–isolate organizational framework of motor cortex.

**Figure 2:**
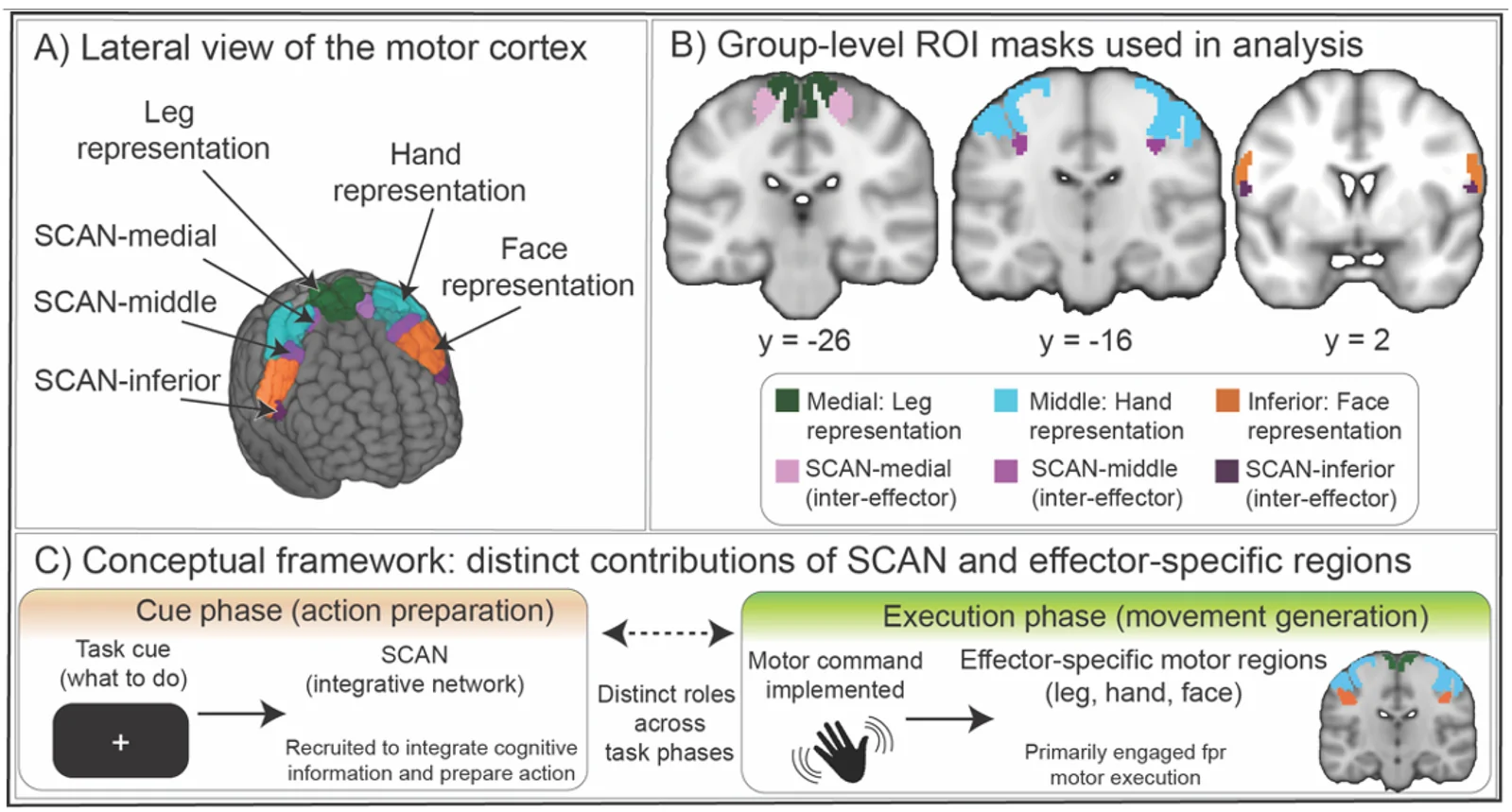
Definition of SCAN regions, effector-specific motor representations, and conceptual framework. (A) Lateral view of the motor cortex showing the spatial relationship between the somato-cognitive action network (SCAN) subdivisions (medial, middle, and inferior) and neighboring effector-specific motor representations corresponding to the leg, hand, and face. (B) Representative group-level ROI masks used in the analyses, displayed on coronal slices showing the leg representation and medial SCAN (y = −26), hand representation and middle SCAN (y = −16), and face representation and inferior SCAN (y = 2). (C) Conceptual framework illustrating the proposed contributions of SCAN and effector-specific motor regions across task phases.

Overview: We combined task-based functional MRI analyses with anatomically defined cortical regions to investigate the spatial organization and functional role of the somato-cognitive action network (SCAN) during motor behavior (Fig. 1). SCAN and effector-specific regions (leg, hand, and face representations) were defined using cortical regions identified in prior studies of motor cortex organization (Gordon, Chauvin et al. 2023). As illustrated in Fig. 2A–B, SCAN regions are positioned between the classical leg, hand, and face representations and were further subdivided into medial, middle, and inferior nodes to assess potential spatial variation in activation. To quantify functional responses, we extracted task-evoked BOLD signals from each region of interest for all participants. Individual responses to motor task conditions were modeled using a general linear model (GLM), enabling estimation of condition-specific activation during both cue and execution phases. Contrast parameter estimates (COPEs) were computed at each voxel to capture differences between task conditions and baseline, and these values were averaged within each region to obtain mean activation values. This framework allowed us to systematically evaluate (i) phase-specific activation differences (cue vs execution), (ii) effector-specific responses during motor tasks, and (iii) differences in activation across the three SCAN subdivisions (medial, middle, and inferior), enabling assessment of spatial gradients within SCAN. In particular, this approach enabled direct comparison between SCAN regions located between classical motor representations and neighboring leg, hand, and face motor regions, providing insight into how activation patterns differ across task phase and cortical location.

### 2.1 Study Population

This study utilized MRI data from the Human Connectome Project (HCP) S1200 release (Van Essen, Smith et al. 2013), which provides extensive neuroimaging and behavioral data from a large cohort of healthy young adults. From the full dataset of 1,206 participants, we included individuals with complete task-based (motor) fMRI data. To ensure a healthy control sample, participants were excluded if they reported any history of illicit or addictive substance use (including cocaine, hallucinogens, cannabis, nicotine, opiates, sedatives, or stimulants) based on HCP screening. Individuals meeting DSM-5 criteria for Alcohol Use Disorder (abuse or dependence), or those reporting an average alcohol consumption exceeding four drinks per week in the year prior to scanning, were also excluded.

The final sample consisted of 701 participants (mean age = 29.3 years, SD = 3.8), including 409 females and 292 males. Handedness was assessed using the Edinburgh Handedness Inventory, with 640 right-handed and 61 left-handed individuals included

### 2.2 MRI Acquisition Protocols

Task-based functional MRI (tfMRI) data were obtained from the Human Connectome Project (HCP) S1200 release, which employed standardized imaging protocols across multiple sites using 3T scanners. Functional images were acquired using a multiband gradient-echo echo-planar imaging (EPI) sequence. Imaging parameters were as follows: repetition time (TR) = 720 ms, echo time (TE) = 33.1 ms, flip angle = 52°, field of view (FOV) = 208 × 180 mm, matrix size = 104 × 90, and 72 axial slices with a multiband acceleration factor of 8. Echo spacing was 0.58 ms, and bandwidth was 2290 Hz/pixel. The resulting spatial resolution was 2.0 mm isotropic. Each motor task session included two runs of 284 volumes, with each run lasting approximately 3 minutes and 34 seconds. The combination of short TR and multiband acquisition enabled high temporal resolution for capturing task-evoked BOLD responses during both cue and execution phases.

#### 2.2.1 Motor-Task Design

The motor task paradigm was adapted from established protocols developed by Buckner and colleagues (Buckner, Krienen et al. 2011, Yeo, Krienen et al. 2011) to elicit robust activation in motor and somatosensory cortical and subcortical regions. Participants were visually instructed to perform movements involving a single body part at a time. In separate blocks, participants executed left or right finger tapping, left or right toe movements, and tongue movements. Each block began with a 3-second cue period, allowing participants to prepare for the upcoming movement, followed by 12 seconds of task execution consisting of discrete, repetitive movements. Each run included 10 movement blocks: two tongue blocks, four hand blocks (two left, two right), and four-foot blocks (two left, two right).

In addition, three 15-second fixation periods were interspersed within each run and served as baseline conditions. During these intervals, participants fixated on a centrally presented cross without performing any movement. All task-related activity was modeled relative to the average signal during these fixation blocks, providing a stable baseline for comparison with movement- and cue-related activation.

### 2.3 fMRI Preprocessing

We utilized minimally preprocessed motor task fMRI data from the Human Connectome Project (HCP) (Glasser, Sotiropoulos et al. 2013). The HCP preprocessing pipeline includes correction for gradient-induced distortions, head motion correction through volume realignment, and EPI distortion correction using spin–echo field maps. Functional images were subsequently registered to each subject’s T1-weighted structural image and normalized to MNI space using the fMRIVolume pipeline. In addition, we applied ICA-FIX denoising to remove structured noise components, including those related to motion. No further preprocessing steps, such as temporal filtering or spatial smoothing, were performed prior to first-level analysis.

To evaluate residual head motion, framewise displacement (FD) (Power, Barnes et al. 2012) was computed using the HCP-provided motion parameters for both left–right (LR) and right–left (RL) motor runs. The average FD across subjects was low (mean FD = 0.18 mm), indicating minimal motion in the dataset. Given the low overall motion and the denoising procedures already incorporated in the HCP pipeline, no additional exclusion criteria based on FD were applied.

### 2.4 Definition and Conceptual Basis of the Somato-Cognitive Action Network (SCAN)

The SCAN was defined based on prior work by Gordon and colleagues (Gordon, Chauvin et al. 2023), who identified inter-effector regions within the primary motor cortex that were functionally distinct from classical effector-specific representations, defined as cortical regions selectively activated during movements of specific body parts (e.g., leg, hand, and face). In contrast to the traditional homuncular organization, in which discrete cortical areas map onto individual effectors, SCAN regions are situated between these zones along the precentral gyrus. These inter-effector regions are proposed to support integrative aspects of motor control, including action preparation, coordination across multiple body segments, and the regulation of internal physiological states.

Consistent with this framework, in the present study we treated SCAN as an integrative system that operates in parallel with effector-specific motor regions. To determine whether functional engagement varies across its cortical extent, SCAN was subdivided into three spatially distinct regions (medial, middle, and inferior) using a data-driven clustering approach (see Section 2.5). This subdivision enabled us to examine whether the magnitude of SCAN engagement and its relationship to effector-specific regions differ across cortical locations. Together, this framework allowed direct comparison of integrative (SCAN) and effector-specific motor systems, and assessment of how their relative contributions vary across both task phase (cue vs. execution) and cortical position.

### 2.5 Subdivision of the Somato-Cognitive Action Network (SCAN)

To generate SCAN subdivisions for subsequent analyses, voxels within the SCAN mask were partitioned into three clusters using k-means clustering (k = 3, 20 replicates). We performed clustering on voxel spatial coordinates using the y and z dimensions and mapped the resulting clusters back into volumetric space and labeled them according to their anatomical locations: medial, middle, and inferior SCAN subdivisions. We utilized binarized versions of each subdivision mask for all subsequent analyses.

### 2.6 Analysis of task-based functional MRI (tfMRI)

We quantified condition-specific activation patterns associated with each cue and movement type in the motor task paradigm. We conducted first-level statistical modeling using a general linear model (GLM), in which each movement condition (left hand, right hand, left foot, right foot, and tongue) was modeled as a 12-second block based on onset timings provided in the HCP explanatory variable (EV) files.

As both left-to-right (LR) and right-to-left (RL) phase-encoding runs were available for each participant, we performed a second level within-subject analysis to combine these runs, thereby minimizing potential biases related to acquisition direction. For each condition, we quantified task-evoked activation using contrast of parameter estimate (COPE) images and extracted mean activation values from predefined regions of interest, including SCAN and effector-specific cortical areas.

The resulting activation values represented COPE estimates derived from subject-level GLMs and were reported in arbitrary units (AU), reflecting the relative magnitude of BOLD signal changes associated with each condition.

Unlike resting-state functional connectivity analyses, which capture spontaneous correlations across distributed networks, this task-based approach focused on temporally locked, condition-specific responses. This enabled us to directly assess how SCAN and effector-specific regions were differentially engaged during distinct phases of motor behavior, particularly during cue-related preparation and movement execution.

### 2.7 Statistical Analysis

Statistical analyses were performed using two-tailed, paired-samples t-tests to assess differences in activation across task phases (cue vs. execution), SCAN subdivisions (medial, middle, inferior), and functional systems (SCAN vs. effector-specific regions).

To control for multiple comparisons, we applied the Benjamini–Hochberg (BH) procedure (Benjamini and Hochberg 1995) across each family of related tests. We defined test families according to the primary hypothesis addressed in each analysis, including phase-related comparisons, SCAN subdivision comparisons, effector-specific regional comparisons, and SCAN–effector interaction analyses. For analyses of effector-specific motor regions, the 10 planned pairwise comparisons across motor tasks were treated as a single family of tests. Within each family, a false discovery rate (FDR) threshold of Q = 0.05 was applied. Corrected significance thresholds are reported in the Results.

## 3 Results

The final study cohort included 701 healthy adults (mean age = 29.3 years, SD = 3.8), comprising 409 females and 292 males. Participants were primarily excluded due to incomplete task-fMRI data or self-reported illicit substance or alcohol use. This cohort partially overlapped with our previously reported resting-state sample (Sadiq, Funk and Waugh 2025, Sadiq and Waugh 2025, Sadiq and Waugh 2026), with 674 participants (approximately 70%) included in both analyses.

### 3.1 SCAN Is Preferentially Engaged During Cue Phase

**Figure 3:**
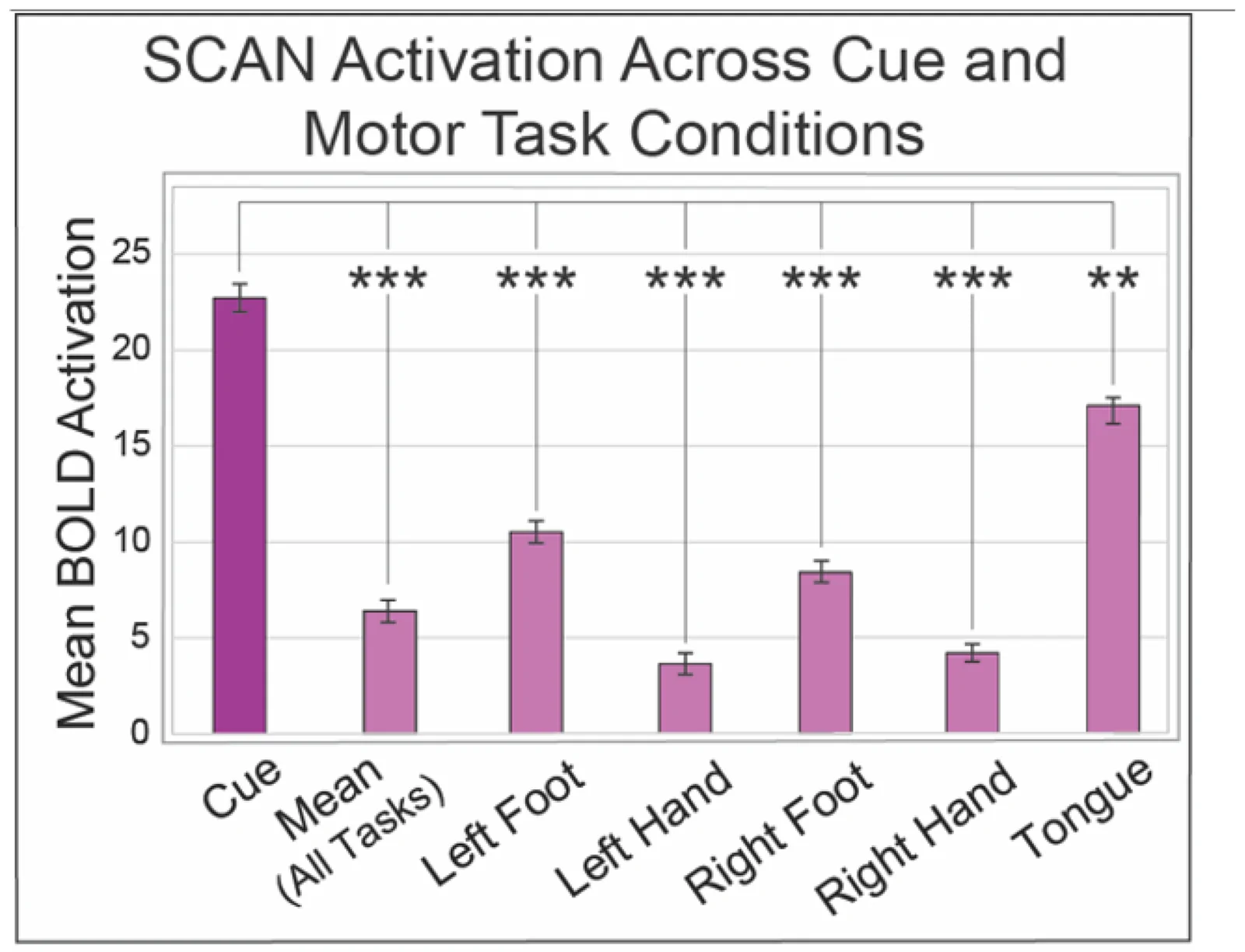
Activation of the somato-cognitive action network (SCAN) across cue and motor task conditions. Mean BOLD activation within SCAN is shown for cue presentation, the average of all motor task conditions [Mean (All Tasks)] and for execution of specific movements (left foot, left hand, right foot, right hand, and tongue). SCAN exhibited significantly greater activation during the cue phase compared to all motor execution conditions, demonstrating that its activity was not driven by effector-specific motor execution. Error bars represent the SEM. **, p < 10^−10^; ***, p< 10^−50^, FDR corrected.

To establish the functional role of SCAN, we first asked whether it exhibited a pattern of activity similar to that of a network known to support action preparation. As a positive control, we compared SCAN with the cingulo-opercular network (CON), an established cognitive control network that maintains task set and supports goal-directed action throughout task performance (Dosenbach, Fair et al. 2008). Consistent with this expectation, SCAN showed significantly greater activation during the cue than execution phase, indicating a primary role in action preparation rather than movement execution. Specifically, mean BOLD activation during the cue phase was substantially higher than during execution (cue: 22.7 ± 0.7; execution: 6.7 ± 0.4; *p* = 1.8 × 10^−100^), corresponding to an approximate 70.6% reduction from cue to execution (Fig. 3). Similarly, the CON exhibited greater cue-related than execution-related activity across all motor conditions, corresponding to an approximate 70.7% reduction from cue to execution (Supplementary Figure S1), supporting the interpretation that SCAN is preferentially involved in action preparation.

### 3.2 SCAN Activity Is Not Driven by Individual Motor-Task Execution

We next examined SCAN activation across individual motor tasks (left-foot, right-foot, left-hand, right-hand and tongue) and found that cue-related activity exceeded execution-related activity for all individual motor-task movements (Fig. 3). SCAN activity during the cue phase (22.7 ± 0.7) was significantly greater than during left foot movement (10.5 ± 0.6; *p* = 3.1x10^−58^), left hand movement (3.6 ± 0.4; *p* = 1.1x10^−97^), right foot movement (8.5 ± 0.6; *p* = 4.2x10^−80^), right hand movement (4.2 ± 0.5; *p* = 8.5x10^−96^), and tongue movement (16.9 ± 0.7; *p* = 1.1x10^−13^). All comparisons survived FDR correction. These differences corresponded to reductions of 53.7% (left foot), 84.1% (left hand), 62.5% (right foot), 81.5% (right hand), and 25.6% (tongue) relative to cue-related activity.

### 3.3 Classical Effector-Specific Somatotopy Is Preserved in Motor Cortex

**Figure 4:**
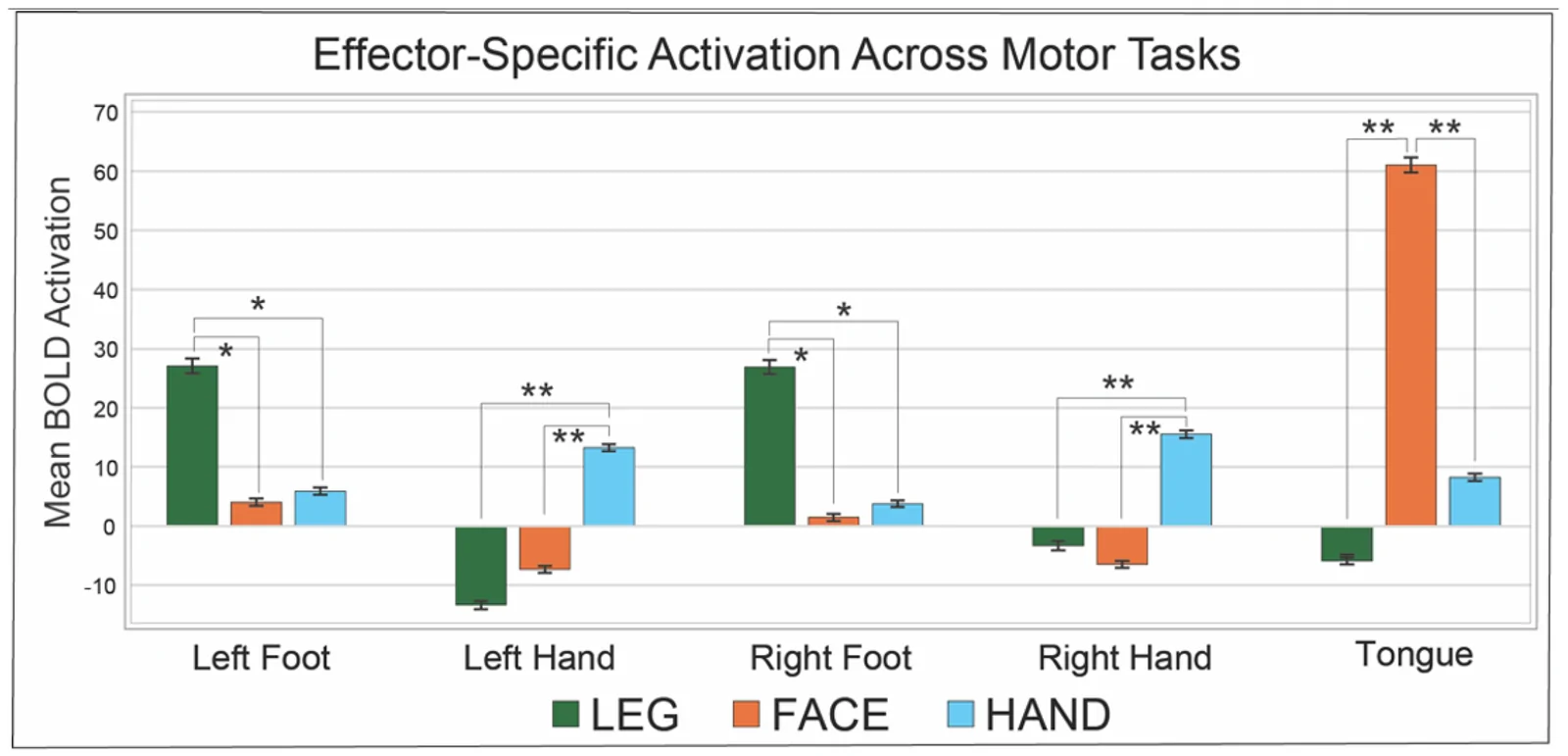
Mean BOLD activation within effector-specific regions (LEG, HAND, and FACE) was correlated with specific motor tasks (left foot, left hand, right foot, right hand, and tongue). Each effector region exhibited maximal activation for its corresponding movement, demonstrating clear somatotopic selectivity (e.g., LEG regions for foot movements, HAND regions for hand movements, and FACE regions for tongue movements). In contrast, non-corresponding effectors showed minimal or negative activation, indicating specificity of these responses. Error bars represent the SEM. **, p < 10^−60^; ***, p < 10^−100^, FDR corrected.

To determine whether SCAN exhibits a distinct functional profile distinct from classical effector-specific motor regions, we first characterized activation patterns within classical effector-specific motor regions (leg, hand, and face) and observed clear somatotopic selectivity, with each region showing maximal activation for its corresponding movement (Fig. 4).

Specifically, LEG regions showed significantly greater activation during foot movements (left foot: 27.1 ± 1.3; right foot: 26.8 ± 1.2) compared to HAND and FACE regions during foot movements **(left foot**: LEG vs. FACE, *p* = 2.5x10^−68^; LEG vs. HAND, *p* = 2.7x10^−75^ ; **right foot**: LEG vs. FACE, *p* = 1.9x10^−83^; LEG vs. HAND, *p* = 1.2x10^−91^). HAND regions exhibited maximal activation during hand movements (left hand: 13.3 ± 0.6; right hand: 15.5 ± 0.6), significantly exceeding activation to LEG and FACE regions (**left hand**: HAND vs. FACE, *p* = 3.6x10^−151^; HAND vs. LEG, *p* = 3x10^−200^; **right hand**, HAND vs. FACE, *p* = 8.1x10^−146^; HAND vs. LEG, *p* = 3.7x10^−117^). Similarly, FACE regions demonstrated the strongest activation during tongue movements (61 ± 1.3), which was significantly higher than LEG and HAND regions: FACE vs. LEG, *p* = 3.3x10^−204^; FACE vs. HAND, *p* = 1.1x10^−204^.

In contrast, non-corresponding effectors (e.g., LEG activation during hand tasks and FACE activation during limb movements) showed minimal or negative activation, further reinforcing the specificity of these responses. Together, these results confirm that classical effector-specific somatotopy is preserved, with each motor region selectively engaged during its corresponding movement.

### 3.4 SCAN Subdivision Activation Increases from Medial to Inferior During Cue

**Figure 5:**
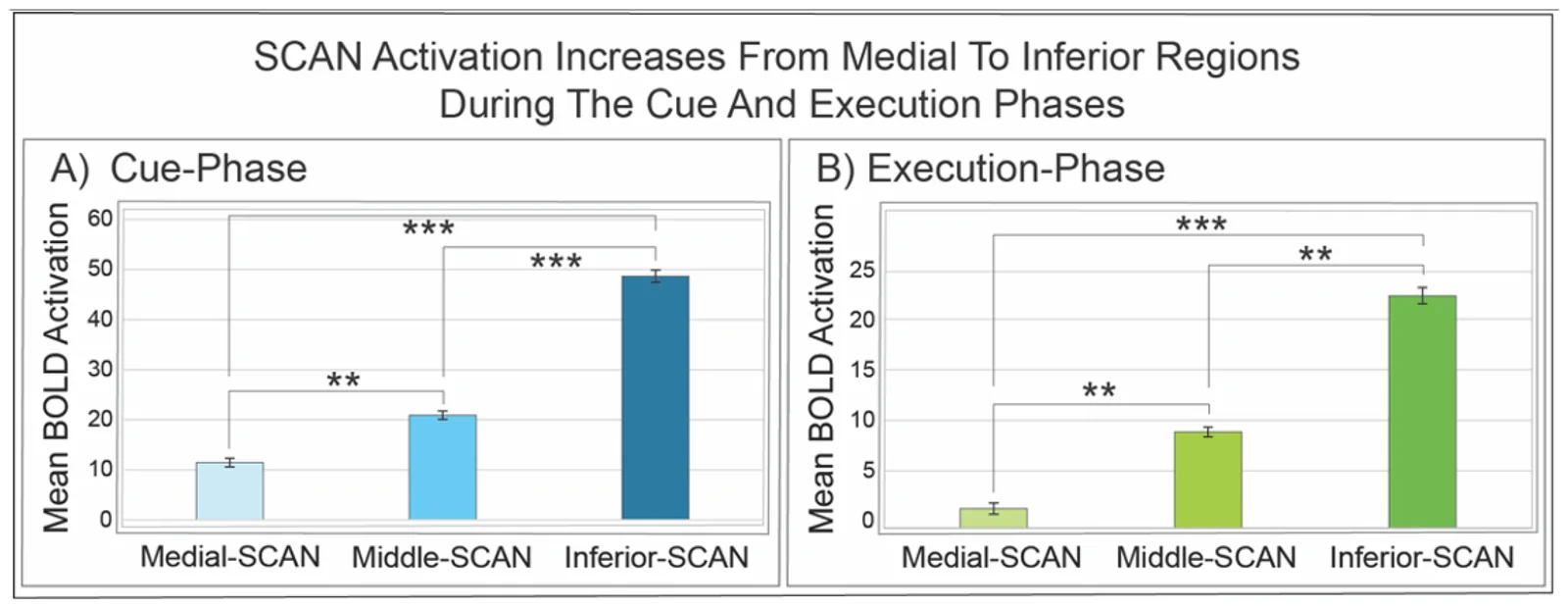
Mean BOLD activation in the medial, middle, and inferior SCAN subdivisions during the (A) cue and (B) motor execution phases. In both task phases, SCAN activation increased significantly from medial to middle and from middle to inferior regions, demonstrating a graded spatial organization along the cortical axis. Although overall activation was greater during the cue phase, the same spatial gradient was preserved during execution. Error bars represent the SEM. **, p < 10^−30^; ***, p < 10^−50^, FDR corrected.

Having established that SCAN differs from classical effector-specific motor regions, we next tested whether the three SCAN regions were functionally homogeneous. We found a graded pattern of activation across its dorsomedial–ventrolateral extent during the cue phase, increasing from medial, to middle, to inferior regions (medial: 11.5 ± 0.8; middle: 20.9 ± 0.8; inferior: 48.7 ± 1.2; Fig. 5A). Comparisons between adjacent subdivisions showed greater activation in middle than in medial SCAN (p = 2.0×10^−25^) and greater activation in inferior than in middle SCAN (p = 5.6×10^−99^). structured spatial organization along the dorsomedial-to-ventrolateral axis. Notably, inferior SCAN showed markedly greater activation compared to other subdivisions, suggesting that engagement of SCAN during action preparation is not evenly distributed across the network. These findings support the presence of functional heterogeneity within SCAN and suggest that its subdivisions may contribute differently to preparatory motor processes.

### 3.5 The Spatial Organization of SCAN Persists During Motor Execution

To determine whether the spatial organization observed during the cue phase was also present during movement execution, we examined activation across SCAN subdivisions. We observed a graded increase from dorsomedial to ventrolateral regions during execution, similar to that observed during the cue phase. Specifically, activation within SCAN increased from medial to inferior regions: medial, 1.8 ± 0.5; middle, 8.9 ± 0.5; inferior, 21.5 ± 0.7; Fig. 5B. Comparisons between adjacent subdivisions showed greater activation in middle than medial SCAN (p = 9.6×10^−35^) and greater activation in inferior than in middle SCAN (p = 2.3×10^−58^). All pairwise comparisons between SCAN subdivisions survived FDR correction.

Although overall activation levels were lower during execution compared to the cue phase, the relative ordering of SCAN subdivisions remained consistent. Together, these findings indicate that the medial-to-inferior organization observed during the cue phase was also present during movement execution, suggesting that this spatial pattern is not limited to preparatory processing alone.

### 3.6 Comparison of Effector-Specific and SCAN Activity Across the Cortical Axis

**Figure 6:**
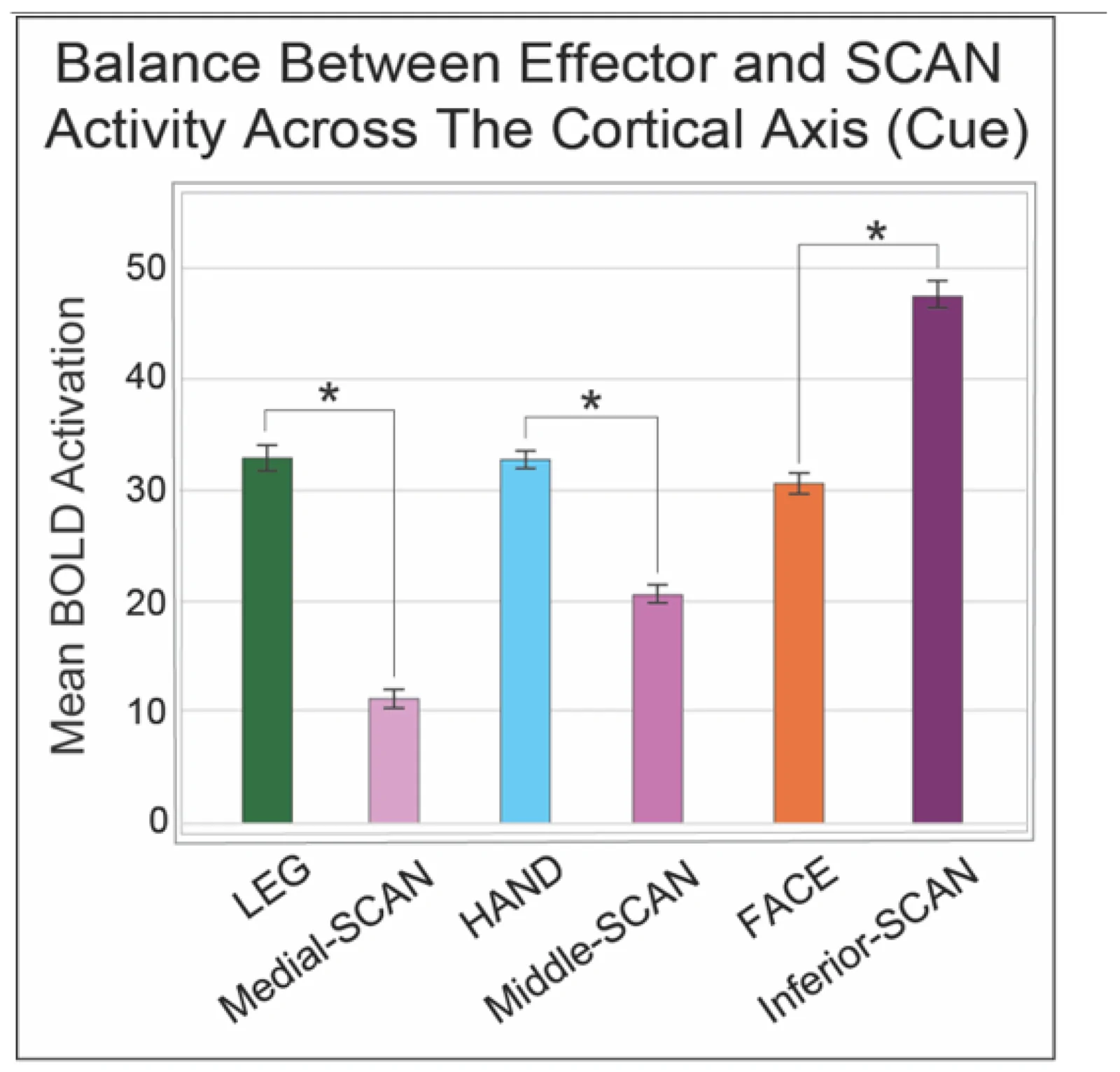
Relative activation of effector-specific regions (LEG, HAND, FACE) and SCAN subdivisions (medial, middle, inferior) across the cortical axis during the cue phase. In medial and middle regions, effector-specific activity exceeded SCAN activity, indicating dominance of effector-specific processing. In contrast, inferior SCAN exhibited significantly greater activation than the corresponding face region, reflecting a progressive shift toward integrative processing as one moves from medial to inferior SCAN subdivisions. These results demonstrate that the relative activation of effector-specific and SCAN regions varies predictably along the dorsomedial-to-ventrolateral cortical axis. Error bars represent the SEM. *, p < 10^−50^; FDR corrected.

Finally, we directly compared effector-specific regions with neighboring SCAN subdivisions and found that the relative dominance of integrative and effector-specific systems varies predictably along the medial–lateral axis of the motor cortex (Fig. 6). In medial regions, effector-specific activity exceeded SCAN activity, with LEG regions showing a 65.7% greater activation compared to medial SCAN (LEG: 33.6 ± 1.2, medial-SCAN: 11.5 ± 0.8; *p* = 5.2x10^−93^). A similar pattern was observed in middle regions, where HAND regions exhibited a 37.6% greater activation than middle SCAN (HAND: 33.5 ± 0.8, middle-SCAN: 20.9 ± 0.8; *p* = 7.4x10^−87^). In contrast, this relationship was reversed in inferior regions, where SCAN activity exceeded effector-specific responses. Inferior SCAN showed a 55.1% greater activation than FACE regions (Inferior-scan: 48.7 ± 1.2, Face: 31.4 ± 0.9; *p* = 7.5x10^−56^).

Notably, neighboring effector-specific regions exhibited relatively similar levels of cue-related activation (LEG: 33.6, HAND: 33.5, FACE: 31.4), whereas SCAN subdivisions showed pronounced spatial variation (medial: 11.5, middle: 20.9, inferior: 48.7). Activation did not differ between leg and hand representations (p = 0.87), whereas face representations showed slightly lower activation than both leg (−7.0%, p = 0.028) and hand representations (−6.7%, p = 0.001). Unlike SCAN, effector-specific regions did not exhibit a medial-to-inferior gradient. These findings indicate that the graded activation profile observed across SCAN is not driven by activation in the adjacent effector regions but reflects a distinct organizational feature of the SCAN network.

## 4 Discussion

Differences in cytoarchitecture, connectivity, and functional engagement suggest that the primary motor cortex is organized according to multiple overlapping principles beyond classical somatotopy (Penfield and Boldrey 1937, Dum and Strick 1991, Graziano 2006). While the traditional homuncular model describes a continuous mapping of body parts along the precentral gyrus, accumulating evidence from non-human primates indicates that motor cortex also contains higher-order organizational features, including distinct anterior–posterior gradients in corticospinal projections and functional zones supporting coordinated, goal-directed movements (Graziano, Taylor and Moore 2002, Rathelot and Strick 2009). These findings suggest that motor cortex may be structured not only by effector-specific representations but also by systems that support the integration of action with behavioral goals. In humans, evidence for such integrative organization has largely been inferred from neuroimaging studies demonstrating distinct large-scale functional networks and effector-specific motor circuits identified through precision functional mapping approaches (Laumann, Gordon et al. 2015, Gordon, Laumann et al. 2017). However, these motor representations are typically characterized as relatively segregated systems and do not fully explain how movement-related processes are integrated with higher-order cognitive control networks during behavior. Recent work has begun to identify a somato-cognitive action network (SCAN), a distributed cortical system that appears to link motor and control processes (Gordon, Chauvin et al. 2023). Gordon et al. demonstrated SCAN activation during motor behavior and established its relationship to large-scale cognitive control networks. Building on this emerging framework, we examined SCAN in a substantially larger cohort and tested whether its activity differs across cue-related and execution-related phases of motor behavior. In addition, we investigated whether SCAN exhibits internal spatial organization by examining activation patterns across medial, middle, and inferior subdivisions. These analyses allowed us to evaluate whether SCAN represents a functionally distinct component of motor cortex and whether its engagement varies predictably across both cortical location and behavioral state.

Large-scale functional brain networks are increasingly recognized as internally heterogeneous systems, exhibiting distinct spatial and functional subdivisions (Power, Cohen et al. 2011, Gordon, Laumann et al. 2017). For example, the default mode network comprises subsystems associated with internally directed cognition and memory processes (Andrews-Hanna, Reidler et al. 2010), while the dorsal attention network exhibits distinct functional pathways linking frontal and parietal regions, supporting multiple representations of attentional priorities (Szczepanski, Pinsk et al. 2013). Similarly, the somatomotor network shows clear topographic subdivisions corresponding to different effectors (Kuehn, Dinse et al. 2017). These findings highlight a general organizational principle in which large-scale networks are composed of multiple functionally specialized components rather than uniform systems. By analogy, the somato-cognitive action network (SCAN) may exhibit similar internal structure, with distinct subregions supporting different aspects of action preparation and integration. However, the extent to which SCAN is functionally subdivided, and whether such subdivisions exhibit systematic spatial organization across the cortical axis, remains unknown.

To examine how integrative and movement-specific processes are organized within the cortex, we investigated activity within the somato-cognitive action network (SCAN) during a motor task paradigm that dissociates cue-related and execution-related processes. In this paradigm, participants were presented with task cues indicating the required response, followed by a distinct execution phase, permitting the temporal dissociation of preparatory and movement-related activity. This approach provides a framework to assess how cortical systems differentially contribute to action planning and execution. Building on our prior work demonstrating dissociable patterns of activation during motor preparation and execution (Sadiq and Waugh 2026), this paradigm enables us to test whether SCAN exhibits similar phase-dependent engagement and whether such effects vary across its spatial extent.

It is important to acknowledge several limitations of this study. First, SCAN was defined using functional criteria derived from task-based fMRI and thus does not correspond to a known anatomical or cytoarchitectonic boundary within the peri-Rolandic cortex. Consequently, the interpretation of SCAN as a distinct functional system is contingent upon the validity and stability of these functional definitions. Although prior work has demonstrated reliable identification of large-scale functional networks using similar approaches, the precise anatomical substrates underlying SCAN remain to be established. Second, the temporal resolution of fMRI limits the extent to which cue-related and execution-related processes can be fully dissociated, and some overlap between preparatory and movement-related activity is likely. While our task design minimizes this overlap by separating cue and execution phases, we cannot exclude the possibility that residual temporal blurring influences the observed activation patterns. Third, our subdivision of SCAN into medial, middle, and inferior components is based on spatial parcellation along the cortical axis and does not necessarily reflect discrete anatomical boundaries. Although the observed gradients suggest systematic functional organization, the extent to which these subdivisions correspond to distinct underlying circuits remains unclear. Finally, the present study focuses on a specific motor paradigm, and the generalizability of these findings to other task domains or behavioral contexts requires further investigation. Future studies combining higher temporal resolution methods, individualized mapping approaches, and multimodal imaging may help to more precisely define the functional and anatomical organization of SCAN.

An additional limitation relates to the definition of SCAN itself. Prior work by Gordon et al. demonstrated that SCAN can be identified at the individual-subject level and exhibits appreciable spatial variability across participants. In the present study, we utilized group-derived consensus SCAN regions rather than individualized functional maps. As a result, the assigned SCAN regions may not precisely correspond to the ideal SCAN location in every participant. Although the large sample size likely mitigates this variability at the group level, individualized mapping approaches may provide a more accurate characterization of SCAN organization and function in future studies.

The present study provides evidence that SCAN represents a functionally distinct system within the primary motor and sensory cortices, characterized by preferential engagement during action preparation and a systematic spatial organization across the cortical axis (Fig. 1). In contrast to classical effector-specific motor regions, which were predominantly engaged during movement execution (Fig. 2), SCAN activity was strongest during cue-related phases, suggesting a role in integrating task goals with upcoming actions. Together, these findings extend current models of motor cortex organization beyond somatotopy, highlighting the presence of integrative systems that support goal-directed behavior. A central finding of this study is the temporal dissociation between SCAN and effector-specific regions. SCAN exhibited preferential activation during the cue phase, whereas effector-specific regions were more strongly engaged during movement execution. Importantly, SCAN activity was elevated during the cue phase relative not only to overall movement execution, but also to each individual motor task. This pattern indicates that SCAN is engaged during action preparation across all motor conditions examined, consistent with a role in integrating task goals and preparing upcoming actions before movement is initiated. This dissociation suggests that SCAN may support preparatory processes, including postural stabilization and the transformation of task instructions into action-relevant representations, while effector-specific regions mediate the implementation of motor output. These findings support a division of labor within motor cortex, in which integrative and execution-related processes are functionally segregated across distinct systems.

This interpretation is further supported by supplementary analyses of the cingulo-opercular network (CON), a cognitive control network previously shown to be functionally associated with SCAN (Gordon, Chauvin et al. 2023). In supplementary analyses, CON activation showed a similar enhancement during the cue phase relative to movement execution, with cue-related activity exceeding all individual motor task conditions. This parallel activation profile supports the interpretation that SCAN participates in action-preparatory processes associated with cognitive control rather than motor output generation alone.

In addition to its temporal profile, SCAN exhibited a graded spatial organization, with activation increasing systematically from medial to inferior cortex (Figs. 5, 6). This gradient was accompanied by a shifting balance between SCAN and effector-specific activity, suggesting that different regions along the cortical axis differentially support integrative versus movement-related processes. Rather than forming discrete, sharply bounded regions, these findings point to a continuous functional organization in which cognitive and motor contributions vary gradually across cortex. Interestingly, this pattern of organization aligns with a broader principle observed across large-scale brain networks, whereby functional systems exhibit internal heterogeneity and spatial gradients rather than uniform activity profiles. Similar forms of structured organization have been described in other networks, such as the default mode (Andrews-Hanna, Reidler et al. 2010) and dorsal attention networks (Szczepanski, Pinsk et al. 2013), supporting the idea that SCAN represents another instance of a network with internally differentiated functional roles.

Notably, the observed dissociation between preparatory and execution-related activity is consistent with our prior findings demonstrating distinct patterns of striatal compartment engagement during motor preparation and execution (Sadiq and Waugh 2026). The present results extend these observations by showing that such temporal specialization is expressed at the level of large-scale cortical networks and further reveal that this organization is structured along a spatial gradient within SCAN.

In conclusion, these findings support an integrate–isolate framework in which motor cortex organization reflects the interaction between systems that integrate cognitive and behavioral goals and those that implement specific motor outputs. Rather than a purely somatotopic structure, the motor cortex appears to be organized along multiple overlapping dimensions, including functional specialization and spatial gradients, enabling flexible and goal-directed behavior.

## Supplementary Material

This is a list of supplementary files associated with this preprint. Click to download.

- SuppFig1.docx

## Data Availability

All MRI and clinical data are freely available through ConnectomeDB, the BIDS-compliant hub for accessing HCP data: https://www.humanconnectome.org/study/hcp-young-adult/document/1200-subjects-data-release.

## Highlights

- The primary motor cortex contains a somato-cognitive action network (SCAN) that is functionally distinct from classical effector-specific motor representations.
- SCAN shows preferential activation during the cue phase, indicating a role in action preparation rather than movement execution.
- SCAN exhibits a graded spatial organization, with activation increasing from SCAN regions located near the leg representation (medial motor cortex) toward SCAN regions located near the face representation (inferior-lateral motor cortex).
- Classical effector-specific motor regions (hand, foot, and face representations) dominate during movement execution, whereas SCAN contributes to integrative, goal-directed processes.
- These findings support an integrate–isolate framework in which integrative and effector-specific functions are systematically organized across motor cortex.
